# efdm–An R package offering a scenario tool beyond forestry

**DOI:** 10.1371/journal.pone.0264380

**Published:** 2022-08-15

**Authors:** Minna Räty, Mikko Kuronen

**Affiliations:** Natural Resources Institute Finland (Luke), Helsinki, Finland; Qinghai University, CHINA

## Abstract

Scenario tools are widely used to support policymaking and strategic planning. Loss of biodiversity, climate change, and increase in biomass demand ways to project future forest resources considering e.g. various protection schemes, alterations to forest management, and potential threats like pests, wind, and drought. The European Forestry Dynamics Model (EFDM) is an area-based matrix model that can combine all these aspects in a scenario, simulating large-scale impacts. The inputs to the EFDM are the initial forest state and models for management activities such as thinning, felling or other silvicultural treatments. The results can be converted into user-defined outputs like wood volumes, the extent of old forests, dead wood, carbon, or harvest income. We present here a new implementation of the EFDM as an open-source R package. This new implementation enables the development of more complex scenarios than before, including transitions from even-aged forestry to continuous cover forestry, and changes in land use or tree species. Combined with a faster execution speed, the EFDM can now be used as a building block in optimization systems. The new user interface makes the EFDM more approachable and usable, and it can be combined with other models to study the impact of climate change, for example.

## Introduction

Forests are a global focus [1,2]. They offer people food, material, income, and recreation. Humanity is facing increasing demand for biomass while seeking ways to tackle climate change and halt biodiversity loss. For example, 31% of endangered species in Finland live in forests [3]. Scenario tools offer one way to study the impacts of decisions and actions on forest resources. Several scenario tools for projecting future forest resources exist.

Forest scenario tools have been developed for different purposes. They may be designed for either strategic or tactical forest planning. The strategic scenario tools usually produce coarse level estimates which can be used for policy support or long-term planning. For example, estimates of regional future wood supply could be used when deciding on industrial investments. The tactical forest management tools are intended for a shorter time horizon and can be used for tactical planning and making management decisions. The modeled unit in the scenario tools may be an individual tree, a size-class, or a whole stand [4–7]. Of the alternatives, the individual tree models are the most expensive to develop, because they require detailed information on the tree level. As the modeled unit gets larger, the results also get coarser and the possibility of predicting the forests’ inner-stand dynamics in respect to e.g. species composition weakens [8].

Matrix models have been widely used and applied all over the world [8]. These matrix models assume that the forest’s next state does not depend on the previous states, but only on the current state. The forests’ dynamics are limited to a finite set of possible future states, and the transitions between states are controlled by transition probabilities. With each simulation step, the transitions between forest states occur according to the probabilities. The first applications of matrix models in forestry are from the 1960s, e.g. [9], and the latest are recent [10]. They have been used to model both the even-aged and uneven-aged forests, monospecific and mixed-species forests, and for the different modeled units, from a single tree to an entire stand [4,8,11–14].

The first matrix model-based scenario tools in Europe were developed more than 30 years ago [15,16]. The most utilized and well-known application based on the pioneering work is the European Forest Information SCENario (EFISCEN) model [17], which is now also freely available and implemented in Java [18]. EFISCEN is primarily designed for managed even-aged forests and large-scale analysis with an entire stand as a modeled unit, where forests’ dynamics are described by age-volume classes [17]. It has been applied for analyses of forest characteristics, harvested wood and biomass, carbon stocks and ecosystem services, e.g. [19–21].

Similarly to EFISCEN, the European Forestry Dynamics Model (EFDM) is an area-based matrix model using the entire stand as a modeled unit designed for large-scale scenario analysis [22]. The EFDM was developed for the JRC (the European Commission’s Joint Research Centre) for even-aged forests [22] and extended to uneven-aged forests [23]. The first implementations of the EFDM included different versions for even-aged (https://github.com/ec-jrc/efdm) and uneven-aged (https://github.com/ec-jrc/efdm-nea) forests. These were published as R scripts [24] under the European Union Public Licence (EUPLv1.1).

The EFDM concept was tested successfully in several countries and growing conditions, even in short-rotation forests [22,23]. [25] studied the effect of including forest owner behavior in scenarios using the EFDM. [26] observed that considering the timber assortments in the modeling was important when the EFDM was used as the forest resource part in forest sector modeling. In the EU-wide DIABOLO project, the EFDM was used to simulate scenarios for the development of forest resources (growing stock) and future biomass supply until 2040 [27]. [28,29] used the EFDM to model the impacts of changes in forest treatments on the future biomass supply. Furthermore, Lithuania utilized the EFDM when estimating its forest reference level (FRL) for accounting for greenhouse gas emissions from land use, land use change, and forestry (LULUCF) to be used as benchmark for accounting net emissions from the existing forests [30].

Unlike the EFISCEN, the EFDM does not include internal models, which means the data requirements differ: The EFISCEN needs the average growing stock and net current annual increment by age classes on which the volume classification, fixed to 10 volume classes, is based. In the EFDM, the number of classes and their limits are for the user to define; their number is neither fixed nor limited, and the growth is estimated based on the input data in the EFDM. However, the EFDM can also utilize databases consisting of the forest resources and model parameters needed for a model run like EFISCEN [31].

We present here a new implementation of the EFDM as the “efdm” R package (https://CRAN.R-project.org/package=efdm). It retains all the main features of its earlier implementations but is more flexible. More specifically: 1) An EFDM run may include forest types which have a different number of classes of the same variables; 2) dynamics in different forest types may depend on different variables, for instance, between age–volume and stem number–volume classes; and 3) the forest area may change forest type and/or land use during a scenario run. These changes mean there is no longer a need to establish separate runs for even-aged and uneven-aged management regimes—a single project is now possible. In addition, the classification of area into land uses and forest types made in the beginning of a simulation is no longer fixed for the entire run. The forest area may change e.g. between even-aged and uneven-aged forests, but also between other land uses and forest types. The changes are possible for any variable the user has used when defining the forest types and land uses.

The efdm package therefore enables simulations of the land use changes, for example deforestation, and the R environment ensures the connections with other models are kept open. The latter has an advantage over most similar models that have been implemented with different software. This paper’s aim is to demonstrate the efdm R package and especially its new capabilities in compared with the previous implementations. Toy data and the codes for replicating the examples and learning the software are included in the package and are available from CRAN (https://CRAN.R-project.org/package=efdm).

### Design and implementation

Previously, the EFDM was available as a collection of R scripts. The scripts were controlled by a set of small text files defining input data, parameter estimation, scenario run, and output. The new R package provides functions to set up the scenario run. The internals have been rewritten completely, resulting in an execution that is considerably faster.

Next, we will explore the basic components of the model and their estimation. The EFDM is an area-based matrix model in which forests and all datasets used are stratified with a set of factors. The user defines the stratification and prepares the datasets accordingly, before importing them to the EFDM. The basic idea in stratification is to divide the land area under observation into homogenous strata which develop and receive similar management. Factors such as site class and fertility may be fixed, i.e. the expected growth potential, administrative region, or protected vs. production forest. Other factors are more dynamic—for example, age and volume, or volume and stem count classes. Some factors like land use are largely fixed but sometimes change.

The simulation time step length must be the same for all model components and the scenario run will therefore also proceed by these time steps. If the forest’s age is used as a class, its class width is also usually the same.

A typical source of information is a forest inventory based on permanent sample plots. In that case the time between inventories is usually chosen as the simulation time step length. Such data between two consecutive measurements are called pair data. Pair data can be used to estimate growth, management activity probabilities, and the impact of management activity on forests’ dynamic classes. In other words, with the pair data, we can observe changes in the forests, and it can thus be utilized for both estimating the activity probabilities and transitions due to the activities. Alternatively, the activity probabilities and transitions can be generated from other models. The pair will then consist of a measured observation and a modeled observation, and the time step length will be defined by the model used. Simple examples of using models to directly define the transitions are final felling and thinning. The final felling as a clear-cut simply changes the forest’s age, stem count, and volume into the smallest classes, or thinning can reduce the volume by a certain number of volume classes.

Management activity is a broad concept which encompasses e.g., forest treatments like fertilization, harvests, and disturbances like forest fires. It also includes the “no management” i.e., growth without management intervention. In other words, activity is any possible forest development alternative, and the set of alternatives depends on the specific implementation and forest strata. However, when the set of activities has been defined both the management activity probability and corresponding transitions should be defined for all strata even if the activity is not applied in all strata. Similarly to the transitions, the activity probabilities are defined externally and then imported into the EFDM. Their estimation can be based on the pair data or other supporting data like management guidelines or tables or expert knowledge.

In a scenario run, the forest areas move between dynamic classes. A scenario run begins with an initial forest state. The initial state is an area distribution of forests into the strata. In each simulation step: 1) the stratified forest state is divided by the activity probabilities into areas receiving different activities, which is saved as a result for the simulation step; 2) areas change classes according to the management defined transitions, i.e. areas “move” between classes; 3) finally, the areas in the strata are summarized for a new forest state for the next simulation step. The previous points are repeated until the end of the scenario run.

The EFDM is a data-driven model that requires the user to prepare the datasets on which the model is built. Model’s level of detail depends on the available data. However, the information can be of different accuracy for different strata. Everything from field sample plot data to statistics and expert knowledge can by utilized and combined. The EFDM breaks down the area distribution in the strata into management activities for each time step. The user is then free to apply any model to the output data to convert the areas into growing stock, biomass, carbon, dead wood, drain, harvest accumulation by timber assortments, harvest income and costs, etc. Scenarios can thus be analyzed from various economic and ecological perspectives.

The efdm package is freely available as an R package for the R programming language [24] from CRAN (https://CRAN.R-project.org/package=efdm). The current version is 0.2.0. The package implements all the previously available features and several new ones. The package contains a vignette available at CRAN that reproduces the examples in this paper and is the recommended starting point for the interested reader.

The main function of efdm package, which performs the simulation, is

runEFDM(*state0, actprob, activities, n*),

where *state0* and *actprob* are data frames describing the initial forest state of the scenario and the activity probabilities, respectively. The *activities* is a list of management activities, and *n* is the number of simulation steps to run.

Management activities are specified using:

define_activity(*name, dynamicvariables, transprobs*).

The first argument *name* is the name of the activity given by the user and it is used to identify the activity in the activity probabilities. The second argument *dynamicvariables* specifies the dynamic variables affected by the activity, and the last argument *transprobs* gives the transition probabilities for the activity. The transition probabilities are specified using a data frame with similar format as Table 1. Table 1 presents all possible transitions for a forest area in the lowest volume and age class (vol0 = 1 and age0 = 1) for the stratum, because the sum of probabilities (prob) equals to 1. We see that in all cases the forest area ages by a class (age1 = 2), but the volume spreads into 6 classes. Most of the forest area (63%) stays in the initial class vol1 = vol0 = 1.

**Table 1:** Example of transition probabilities (prob) for a forest at Mineral soil in the Middle Boreal vegetation zone with ‘other’ as a dominant tree species in respect to dynamic variables vol and age where index ‘0’ refers to the initial state and ‘1’ to the new states after one simulation step.

vol0	age0	soil	region	sp	vol1	age1	prob
1	1	Mineral	Middle	other	1	2	0.629107981
1	1	Mineral	Middle	other	2	2	0.145539906
1	1	Mineral	Middle	other	3	2	0.122065728
1	1	Mineral	Middle	other	4	2	0.089201878
1	1	Mineral	Middle	other	5	2	0.009389671
1	1	Mineral	Middle	other	6	2	0.004694836

In case pair data (see Table 2) is available estimatetransprobs function can be used to estimate transition probabilities by the methods described in [32].

**Table 2:** A subset of the pair data in the example dataset: The stratifying variables region, soil, dominant tree species (sp) are accompanied by observed change in dynamic variables vol and age, where index ‘0’ refers to the first and ‘1’ to the second measurement of the sample plot.

region	soil	sp	vol0	vol1	age0	age1
South	Mineral	spruce	10	7	13	14
South	Mineral	spruce	8	8	20	21
South	Mineral	other	9	10	5	6
South	Mineral	other	1	1	1	2
South	Mineral	other	7	8	2	3
South	Mineral	other	7	10	5	6
South	Mineral	other	3	5	31	32

## Use examples

The first use case shows a traditional run and the results of the matrix forest model. The second and third use cases demonstrate the new features included in the R package. All datasets and codes for the following use cases and more examples are available as package vignette.

All our examples concern 18.4 million ha of managed forest land in Finland. These forests are presented by volume–age class in strata separated by the dominant species, spruce (*Picea abies*, L.) and others (all other tree species), and soil type, mineral soils and peatlands in three vegetation zones: 1) hemiboreal and southern boreal; 2) middle boreal; and 3) northern boreal. The number of volume and age classes is 15 and 35, respectively. The width of age class is five years, which coincides with the length of the simulation step and inventory interval of the sample plots. The forests receive one of three alternative activities in each 5-year time step: growth; thinning; and final felling. No management activities or treatments are applied to growth. Thinning removes only part of the growing stock and may also change the age class. Final felling is a clear-cut, in which both the volume and age of forest move to the smallest classes. Activity probabilities and transitions by activities have been derived from a pair dataset of consecutive inventories of the sample plots.

### Example 1: Even-aged forest scenario projections

**Fig 1:**
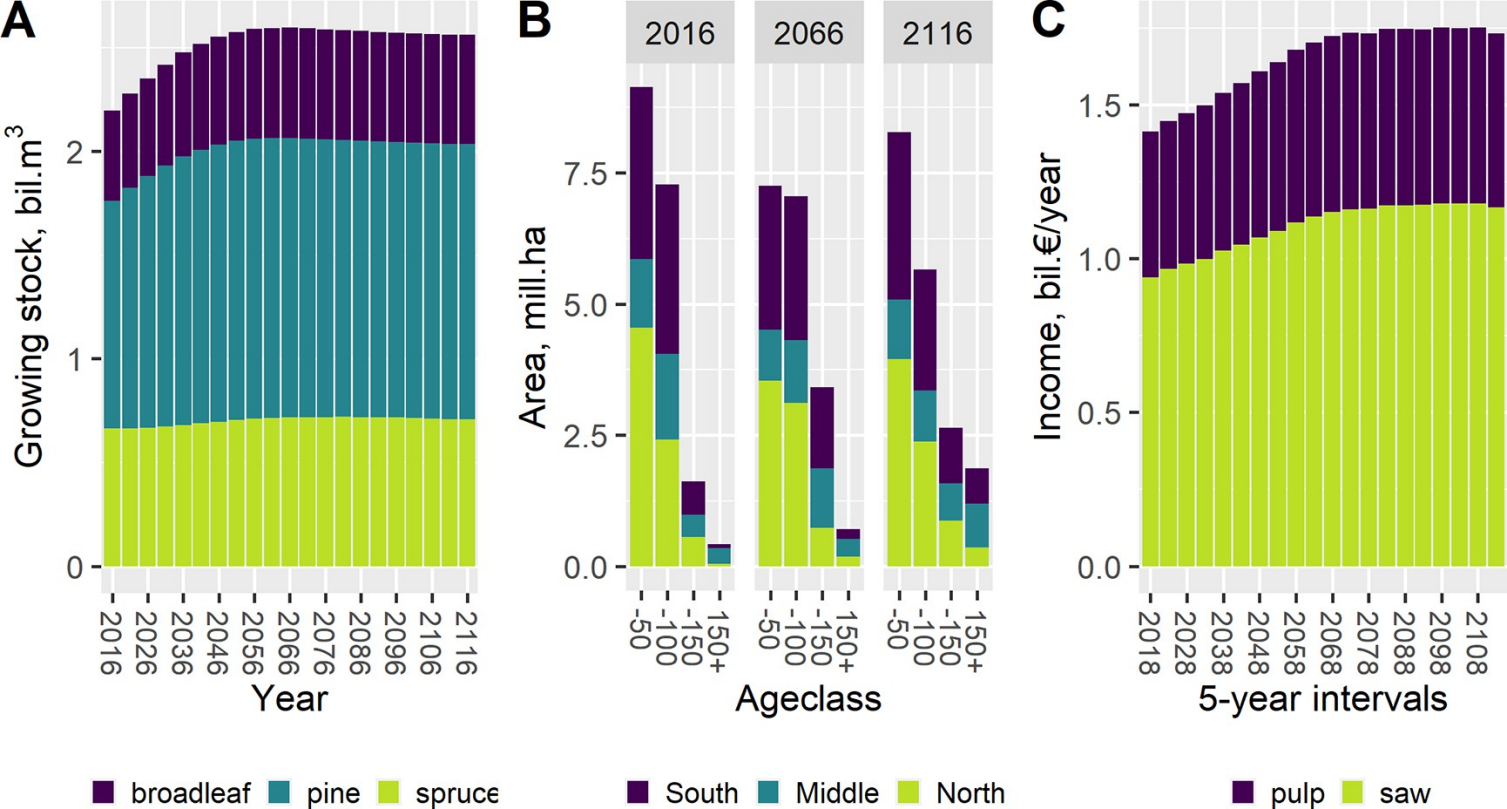
Examples of typical forest scenario results from a 100-year simulation in 5-year time steps: (A) Growing stock; (B) area–age distribution by vegetation zones in 2016, 2066, and 2116; and (C) harvest income by timber assortment.

The model was run for 100 years from 2016, even though 50 years or less (e.g. [33]) is recommended. The results for total growing stock, age distribution, and harvest income were estimated (Fig 1). The total growing stock increased during the first decades, then settled, and even slightly decreased toward the end (Fig 1A). The area of old forests increased in all vegetation zones (Fig 1B). The harvest income followed the development of total growing stock with a delay (Fig 1C).

In our example, the forest area in the largest volume class was increasing. As a forest reaches the largest volume class, it cannot grow due to the classification. The same applies for the age classes in these use cases: The mean age of the largest age class is fixed throughout the simulation and the forest area in the class is therefore not aging. In other words, the chosen classification affects the results and should be noted when interpreting them in respect to the model’s dynamic classes.

The simulation was based on the observed changes in the past, assuming the future forest development and management remained the same for the next 100 years—which is unlikely.

### Example 2: Change in forest strata–dominant tree species change

It is assumed that ongoing climate change will affect future global tree species distribution. According to projections for the Intergovernmental Panel on Climate Change (IPCC) climate scenarios, a strong A1FI, a moderate A1B, and a mild B2 scenario, spruce (*Picea abies*, L.) will move northward in Finland, replacing pine (*Pinus sylvestris*, L.). In contrast, pine will take over from spruce in the south [34,35]. These trends are incorporated in this example through final felling activity. In the hemiboreal and southern boreal vegetation zones, 30%, and in the middle boreal zones, 20%, of previously spruce-dominated forest will move after final felling to another (tree species) dominant strata. Although the percentages are made up for this example, the change is driven by two forces: First, in a warming climate, spruce will suffer from drought on less fertile sites. Second, on fertile sites, change is inevitable due to increased spruce root and butt rot (*Heterobasidion parviporum*), which prevents the planting of spruce. In the northern boreal vegetation zone, forests dominated by other tree species became spruce-dominated after final felling at a rate of 20% of all final felled forests. The transition probabilities were fixed constants in this example, but these could also be derived dynamically from a separate model during simulation. The other parts of the model remained the same as in the first use case above.

**Fig 2:**
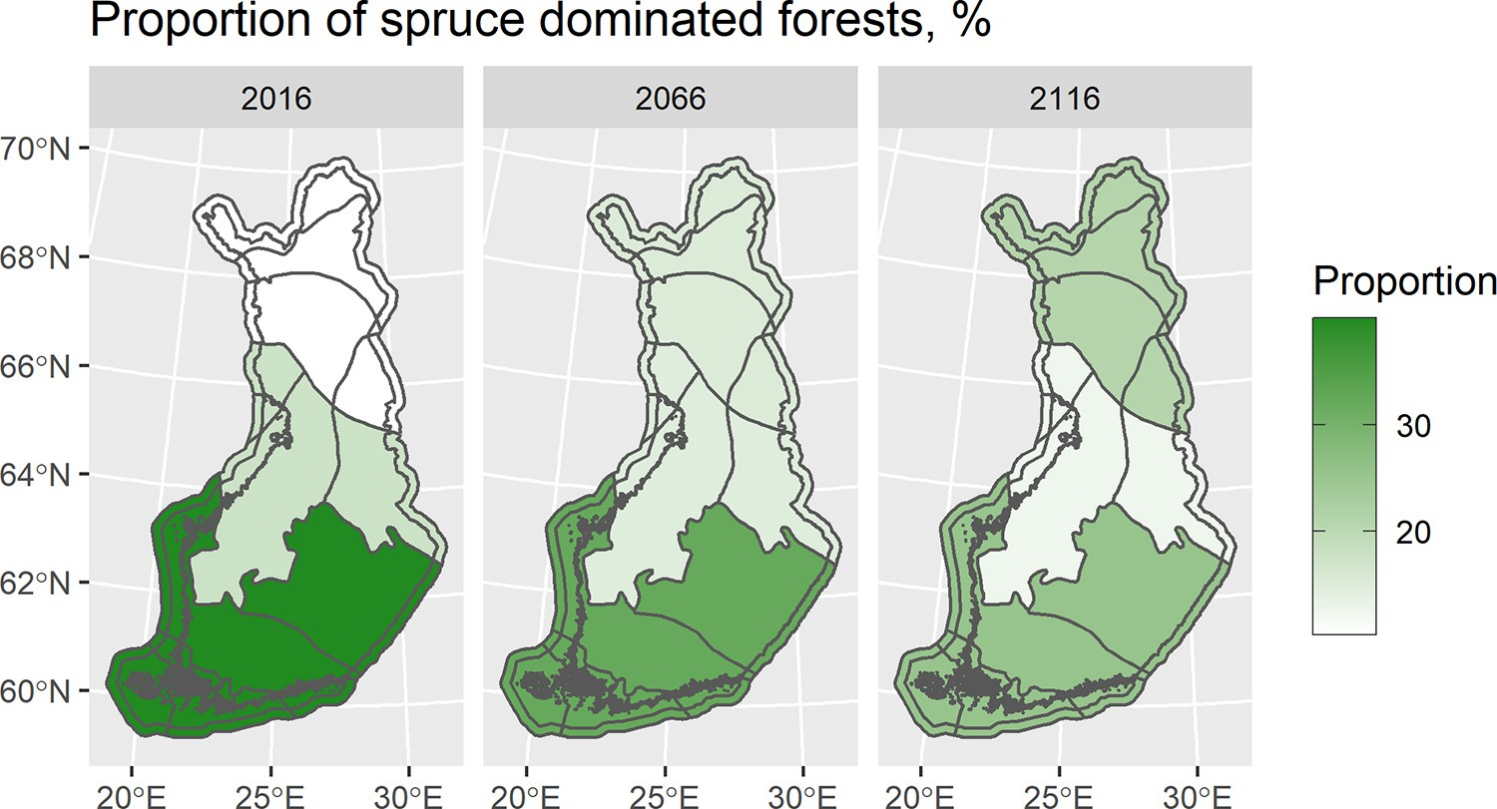
Proportion of spruce (*Picea abies*) -dominated forests in 2016, 2066, and 2116 by vegetation zones when climate change is assumed to affect the future tree species distribution [Bio-geographical regions / Source: Finnish Environment Institute].

The timeseries for the area proportion of spruce dominated forests in vegetation zones in 50-year lags, 2016, 2066, and 2116, show the spruce-dominated forests shifting northward (Fig 2). In the northern boreal vegetation zone, the proportion of spruce-dominated forests doubled in 100 years. In the middle and southern boreal vegetation zone the proportion of spruce-dominated forests decreased by 30% and 36%, respectively. The effects on national forest resources compared to the previous use case are minor. However, a decrease in income is increasing after year 2061, and being highest in the end of simulation period, 4.6% less than in the Example 1 by year 2116.

In the previous version of the EFDM, it was impossible to include changes with respect to any classifying variables. Here, the change was demonstrated with respect to dominant species, but it could happen with any stratifying variables. The change was also linked to an activity, but it could also be an activity of its own (see Example 3) or happen without a link to an activity. In the latter case, the stratum’s transition probabilities should include this probability.

### Example 3: Land use changes

Globally, the forest area and its transitions to and from other land uses have been monitored for decades [2]. According to a new report, deforestation is relatively small in Finland at around 10,000 ha annually, but its impacts on greenhouse gas emissions and Finland’s climate targets are very significant [36]. Afforestation is even smaller than deforestation at around 3,000 ha annually.

Land use changes such as deforestation and afforestation can be included in EFDM analysis. We defined three land use categories: forest, agriculture, and other. The last of these refers to built-up land. In this example, we allowed changes from forests to both other land use categories (Deforestation) and from agricultural land to forests (Afforestation). These land use changes were implemented as a set of three new activities besides growth, thinning, and final felling: 1) the land use change from forest to agricultural land, 2) the land use change from forest to other land use, and 3) the land use change from agricultural land to forest. In addition, a dummy activity was also needed in agricultural and built-up land uses for the areas which did not change the land use category.

**Fig 3:**
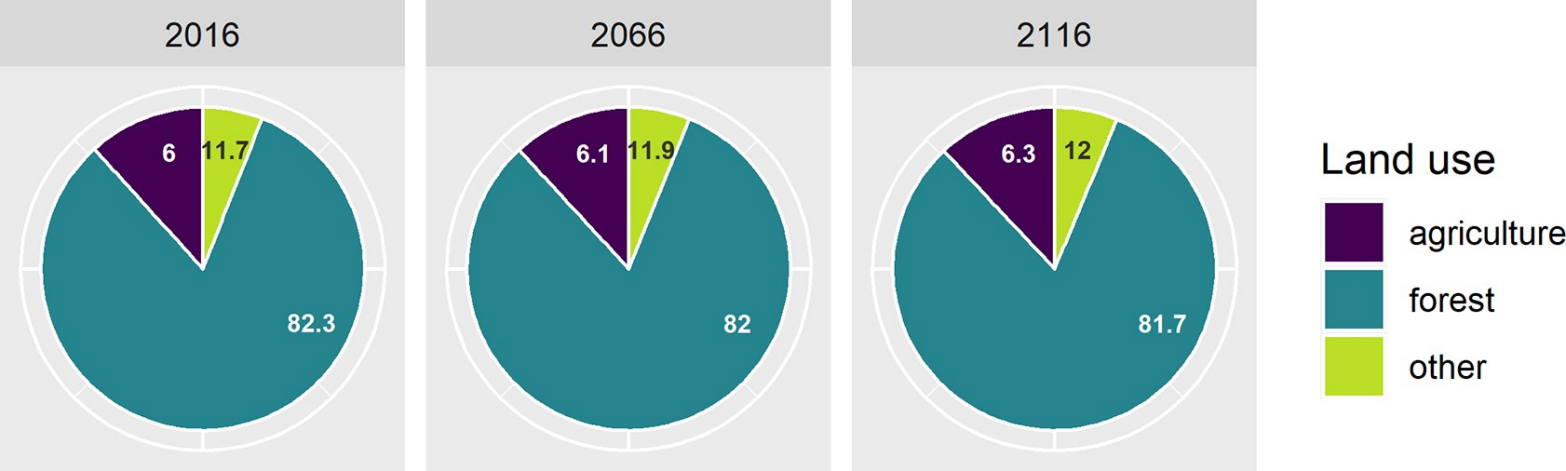
Percentages of different land uses in 2016, 2066, and 2116.

The change rates between the land use categories were implemented as activity probabilities for the above defined new activities. The change rate from forest to agricultural and other land was set at 0.025% and 0.02% respectively, and from agricultural land to forest at 0.05%. We also assumed that the change rates did not depend on region or forest strata, and remained constant over the simulation period. These percentages produced approximately the same net loss of forest land as reported in [36]. The forest area decreased in a 100-year simulation by 0.5% (Fig 3). The changes in forest resources are not shown here, but they could be estimated similarly to previous use cases.

This example enabled us to show how land use changes could be incorporated in the EFDM. We used a very coarse approach, but the EFDM could be coupled with specific models of other land use categories. The change rate between land use categories could also be determined dynamically for each simulation step with a separate model. Such model coupling would lead to the holistic modeling of the bioeconomy.

## Discussion and future directions

The efdm R package provides a fast and flexible implementation of the EFDM with a modern interface. Despite a complete rewrite of the internals, the basic components of the model and the result the user sees remained the same. However, the R package version gives more choice to the user to define scenarios. These new features respond to current and increasing needs in forestry scenario analysis. To our knowledge, the EFDM is the first forest scenario tool distributed as an R package.

As the EFDM offers flexibility for the user to define scenarios and estimate the model components it also leaves the responsibility to the user. The user should be able to compile appropriate datasets for estimating the transitions, decide on the stratification and classification, prepare activity probabilities, initial state, and, if desired, output coefficients for the simulations. All the components listed except the output coefficients are needed for a simulation. Estimating these require deep knowledge of the field and access to forest data, but different data sources can be exploited and combined in the estimation. Decisions made in the processing have an impact on the results. The chosen classification may limit the analysis like in the Example 1 where volume development result may have been affected by the chosen largest classes. Further, an error made in the preparation will propagate to the results.

The EFDM is still a large-scale scenario tool, which cannot reflect inner-stand interactions and dynamics, nor provide information for short-term horizon tactical forest planning. Other software designed particularly for decision support in forest management, such as Heureka [5] or Motti [37], should be considered for these purposes [38]. All simulators discussed in this paper despite the method aim at predicting the future. The predictions may be based on e.g. observed past development, models derived from experimental data, or market forecasts. In that sense all simulations are wrong [39], and therefore the results should be interpreted against the model limitations [33].

In future, we will apply the EFDM to a broad range of research and policy questions. One aim is to undertake analysis beyond forestry—for example, biomass scenarios—to incorporate the effects of climate change in the model. Another study aim is to analyze the impacts of alternative forest treatments, nature conservation management, and protection on both the biodiversity of forests and biomass production, and how these goals, often perceived as contradictory, could be harmonized with different land uses and at the landscape level.

We also hope the R package will be widely distributed, and its user community will grow. This would eventually enable large-scale, even global, analysis and build on the bottom-up approach to which the local forestry experts contribute.
